# Incidence, Risk Factors, and Outcome of Acute Kidney Injury in the Intensive Care Unit: A Single-Center Study from Jordan

**DOI:** 10.1155/2020/8753764

**Published:** 2020-07-30

**Authors:** Ashraf O. Oweis, Sameeha A. Alshelleh, Suleiman M. Momany, Shaher M. Samrah, Basheer Y. Khassawneh, Musa A. K. Al Ali

**Affiliations:** ^1^Department of Internal Medicine, Nephrology Division, Jordan University of Science and Technology, Irbid, Jordan; ^2^Department of Internal Medicine, Nephrology Division, The University of Jordan, Amman, Jordan; ^3^Department of Internal Medicine, Respiratory and Critical Care Division, Jordan University of Science and Technology, Irbid, Jordan

## Abstract

**Background:**

Acute kidney injury (AKI) is a common serious problem affecting critically ill patients in intensive care unit (ICU). It increases their morbidity, mortality, length of ICU stay, and long-term risk of chronic kidney disease (CKD).

**Methods:**

A retrospective study was carried out in a tertiary hospital in Jordan. Medical records of patients admitted to the medical ICU between 2013 and 2015 were reviewed. We aimed to identify the incidence, risk factors, and outcomes of AKI. Acute kidney injury network (AKIN) classification was used to define and stage AKI.

**Results:**

2530 patients were admitted to medical ICU, and the incidence of AKI was 31.6%, mainly in stage 1 (59.4%). In multivariate analysis, increasing age (odds ratio (OR) = 1.2 (95% CI 1.1–1.3), *P* = 0.0001) and higher APACHE II score (OR = 1.5 (95% CI 1.2–1.7), *P* = 0.001) were predictors of AKI, with 20.4% of patients started on hemodialysis. At the time of discharge, 58% of patients with AKI died compared to 51.3% of patients without AKI (*P* = 0.05). 88% of patients with AKIN 3 died by the time of discharge compared to patients with AKIN 2 and 1 (75.3% and 61.2% respectively, *P* = 0.001).

**Conclusion:**

AKI is common in ICU patients, and it increases mortality and morbidity. Close attention for earlier detection and addressing risk factors for AKI is needed to decrease incidence, complications, and mortality.

## 1. Introduction

Acute kidney injury (AKI) is a well-known complication that affects critically ill patients in intensive care unit (ICU) and is associated with increased mortality, morbidity, and length of stay [1]. The incidence of AKI is extremely variable between 2.5% and 92% [2–5], which necessitate earlier detection and management to decrease risk for death, prolonged hospitalization, and future development of chronic kidney disease (CKD) [6, 7]. There is no uniform definition of AKI in the ICU settings. Several studies used different classifications to define AKI in ICU patients [8] including RIFLE (risk, injury, failure, loss, and end-stage renal failure) [9], AKIN (acute kidney injury network) [10], KDIGO (kidney disease improving global outcomes) [11], and CK (creatinine kinetics) [12].

Studies on the incidence of AKI among patients in the ICUs in Jordan and the region are scarce. The aim of this study was to describe the incidence, risk factors, and outcome of AKI in patients who were admitted to the medical ICU in Jordan.

## 2. Materials and Methods

### 2.1. Patient Selection and Data Collection

In a retrospective study design, data were collected from patients who were admitted to the medical ICU at King Abdullah University Hospital (KAUH), a 650-bed, 16 medical ICU beds, urban academic tertiary referral hospital, that serves 5 provinces in the north of Jordan.

We reviewed the medical records for all patients who were admitted to the medical ICU between 2013 and 2015. One admission (the first) was analyzed if the patient had more than one admission.

Demographic data, including age, gender, comorbidities, the cause of admission, medications, laboratory data, and length of ICU stay, were extracted from patients' electronic records. We used (AKIN) [13, 14] classification to define and stage AKI.

AKIN is a modified version of the older RIFLE classification for AKI, which requires an increase in serum creatinine by at least 26.4 *µ*mol/l within a period of 48 hours or by a decrease in urine output; according to AKIN, AKI is considered after achieving adequate hydration and after excluding urinary obstruction. Due to the retrospective nature of the study, data regarding urine output were not available to be included in AKI definition.

The Modification of Diet in Renal Disease (MDRD) equation was used for estimation of glomerular filtration rate (eGFR). Contrast exposure was defined as intravenous contrast administration within one week of AKI onset.

The study was approved by the institutional review board at Jordan University of Science and Technology and King Abdullah University Hospital.

### 2.2. Statistical Analysis

Data analysis was performed using Stata/SE, version 12.1 (StataCorp, College Station, TX). Patients were divided into two groups: patients with and without AKI. For continuous variables, mean, standard deviation (±SD), minimum, and maximum were used, and for differences between normally distributed values, we used the unpaired t-test. Percentages were used for categorical variables. The Mann–Whitney U-test was used to compare nonparametric groups when data were nonnormally distributed. The Pearson chi-square test was used to test categorical variables. Univariate and multivariate regression analyses (based on significant factors in univariate analysis plus those factors considered clinically significant by consultant nephrologist, such as age, gender, comorbidities, APACHE II score, and serum albumin) were performed to determine the independent predictors of AKI.

Kaplan–Meier survival analysis was performed to assess 12 months mortality outcomes.

## 3. Result

Records of 2530 patients were reviewed, and 237 patients were excluded: 174 patients for missing data and 63 patients had end-stage kidney disease and were on chronic hemodialysis.

The mean age for the patients was 54.3 years (range 16–114) and 58% were males.  Table 1 describes their baseline characteristics.

Mean arterial pressure (MAP) at admission (using the average of the first 3 consecutive readings) was 80.3 mmHg for the AKI group and 77.0 mmHg for the non-AKI group (*P* = 0.06). Mean eGFR at admission was 89.2 ml/min (SD ± 71.2): 70.2 ml/min (SD ± 60.5) for the AKI group vs. 98.0 ml/min (SD ± 74.0) for the non-AKI group (*P* < 0.001).

The most common cause of admission was for neurological causes (19.7%) followed by respiratory causes (19.2%) (Table 2). While the commonest cause for AKI was sepsis (29.8%) followed by neurological disorders (18.7%).

**Figure 1:**
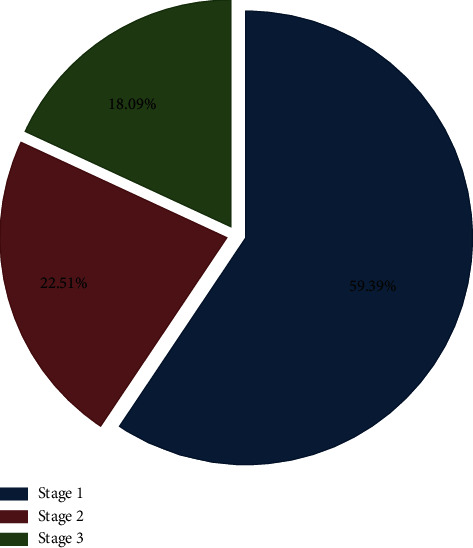
AKI by AKIN stage.

The incidence of AKI was 31.6% and most were in stage 1 AKI (59.4%) (Figure 1).

In patients who developed AKI, 20.4% were started on hemodialysis for different reasons: 15% started on dialysis for persistent hyperkalemia, 46.6% were dialysed for fluid overload not responding to diuretics, and 38.4% started for the combination of both reasons.

**Figure 2:**
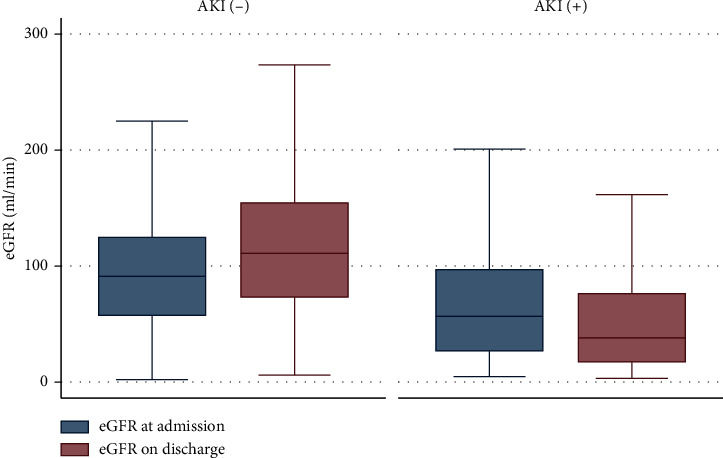
Boxplot graph for eGFR by AKI status.

By the time of discharge, mean eGFR was 106.4 ml/min (SD ± 164.1): 61.0 ml/min (SD ± 83.6) for the AKI group vs. 129.1 ml/min (SD ± 187.9) for the non-AKI group (*P* < 0.001) (Figure 2).

Using univariate analysis, increasing age (*P* < 0.001), hypertension (HTN) (*P* = 0.001), the use of angiotensin converting enzyme inhibitors (ACEI)/angiotensin II receptor blockers (ARBs) (*P* = 0.005), APACHE II score (12.2 (SD ± 4.1) for the AKI group vs. 10.7 (SD ± 3.8) for the non-AKI group ( *P* = 0.0001)), and serum albumin at admission (mean serum albumin for the AKI group was 30.1 g/l (SD ± 9.4) and 33.5 g/l (SD ± 8.9) for the non-AKI group (*P* = 0.001)) were predictors of AKI (Table 3).

In multivariate analysis, age (HR 1.2, 95% CI 1.1–1.3, *P* = 0.0001) and APACHE II score (HR 1.5, 95% CI 1.2–1.7, *P* = 0.001) were strong predictors of AKI. Mean length of ICU stay was 11.5 days, 10.3 days for the AKI group vs. 12.3 days for the non-AKI group (*P* = 0.07). Unfortunately, data regarding duration between admission to ICU and AKI occurrence were not available or accurate to be included in the study.

The overall mortality rate was 38.1%, almost half of the patients who died (55.2%), during their hospitalization.

**Figure 3:**
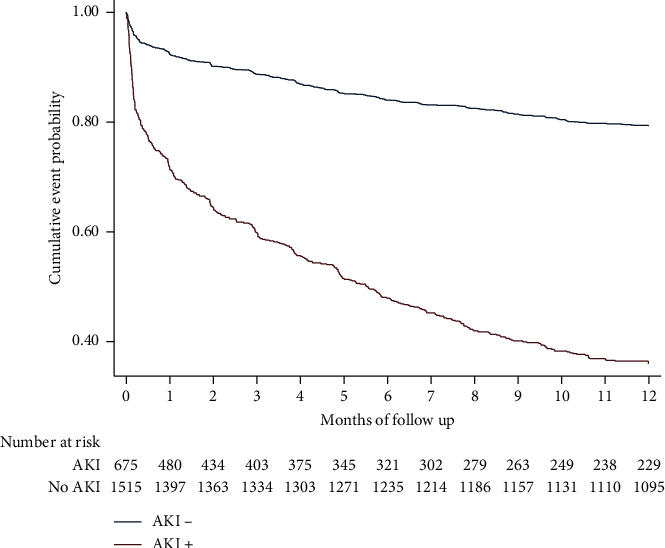
Kaplan–Meier curve for one year survival.

Based on time of discharge, the mortality rate was as follows: at the time of discharge, the mortality rate was 58% for the AKI group vs. 51.3% for the non-AKI group (*P* = 0.05). By 30 days after discharge, the mortality rate was 1.0% for the AKI group vs. 0.4% for the non-AKI group (*P* = 0.03). At one year, the mortality rate was 66.1% for the AKI group vs. 27.7% for the non-AKI group (*P* = 0.001) (Figure 3).

Based on AKI stage, patients with more severe AKI (stage 3) had a higher mortality rate (88%) compared to patients with stage 2 (75.3%) and stage 1 (61.2%) (*P* = 0.001).

## 4. Discussion

AKI is a growing problem worldwide, consuming a lot of resources, and can be variable between high- and low-income countries [15]. Its association with mortality, morbidity, CKD, and prolonged hospitalization especially in critically ill patients and patients admitted to ICU is well documented in the literature, which reflects the worldwide importance of early detection and prevention with the aim of decreasing death and morbidity [16–18].

To our knowledge, our study is the first to discuss incidence and risk factors for AKI in ICU patients in Jordan; the incidence of AKI was 31.6%, and risk factors for developing AKI, in our ICU patients were older age, HTN, use of ACEI/ARBs, and having low serum albumin on admission. More severe critical illness based on higher APACHE II score was a strong predictor for developing AKI.

Studies revealed variable incidence of AKI in ICU patients, ranging from as low as 2% to as high as 92% [3].This variability can be attributed to the use of different classifications RIFLE, AKIN, KDIGO, and CK across studies [3, 19, 20] with or without inclusion of urine output criteria [21–24]. Some of the studies suggested better accuracy when AKIN criteria was used for evaluation of the incidence of AKI, while RIFLE criteria may be better in predicting mortality according to others [19, 25].

In our cohort, the incidence of AKI was 31.6%; this relatively modest incidence can be explained by including a wider spectrum of ICU patients, such as less septic patients and more patients with cardiovascular and neurological illnesses, and not including urine output criteria [24, 26], although as expected sepsis was the commonest cause for AKI, with 29.8% of septic patients developed AKI.

Most of the studies with higher incidence of AKI and mortality were carried out mainly on septic patients [3, 27–33]. A study from Iran (a close by regional country) showed a 37% incidence of AKI using RIFLE criteria, and the causes of admission to ICU were not mentioned, which one may assume that their cohort included more septic patients, and the incidence of AKI in the study was still close to our results [34].Another review article which studied patients from around 300 ICUs worldwide indicated that the incidence of AKI was around 33.4% in developed countries and around 37.7% in developing countries [3].

In our study, most of the patients with AKI were in stage 1 according to AKIN criteria (59.39%). This is consistent with other studies where the majority of patients with AKI were in stage 1. In a large French cohort, 5242 patients were included from 23 ICUs, 2458 patients were in stage 1 (46.89%) [35], and in another Chinese cohort with a total of 3107 patients with AKI, 23.1% of these patients was in stage 1 [4]. A cohort from UK by Zhang et al. which included 2525 patients with AKI in ICU showed around 41.2% of patients was in stage 1 [36].

When comparing patients from our cohort, values of eGFR on admission and on discharge were significantly lower in the AKI group. This may indirectly imply the higher risk of developing CKD in patients with previous AKI, especially with increase of stage and severity of AKI [7]. It appears that developing more severe AKI will increase the risk for developing CKD after hospital discharge, even in less severe AKI, and risk for CKD can be still appreciated and needs more prolonged follow up [7]. Due to the retrospective nature of our study, we could not define the incidence of progression to CKD in patients due to lack of data.

A cohort from 30 ICUs in Beijing showed a mortality rate of 54.50% in patients with AKI and sepsis, and the risk for mortality increased with increasing age, cardiac complications, hypertension, CKD, and higher APACHE II score among other risk factors [29]. In studies of AKI in ICU trauma patients, age, HTN, DM, sepsis, and higher APACHE-II score will increase chance for AKI and complications [37].

Comparing to other studies, 30 days mortality rate was ranging from 22%–52% [2, 38]. In our study, we divided the mortality rate based on the time of discharge. Regardless of the time of discharge, the mortality rate was higher in patients with AKI, having most of the deaths during the hospitalization.

In our previous studies involving AKI in elderly patients, we found that age above 60 years will have a higher risk for AKI, morbidity, mortality, length of hospital stay, and need for RRT [39, 40]. A recent AKI in ICU epidemiology overview by Santos et al. which included data of 67,033 patients from more than 300 ICUs from different regions of the world showed that mortality can range from 5% to 80% (highest) in septic patients, and the need for RRT can range from 0.8% to 59.2% [3]. Comparing patients with AKI and sepsis to patients with AKI due to other causes, as carried out by a prospective cohort by Pinheiro et al., showed that sepsis will cause worse prognosis and higher mortality and morbidity in AKI patients [30]. Less overall mortality and morbidity in AKI patients were noted in ICUs that have fewer septic patients, and makes us conclude that sepsis has a significant contribution to increased mortality rate in ICU-AKI patients, which emphasizes the need for early detection and treatment of AKI in this group. In our cohort, the overall mortality rate in the ICU was 38.1%, with more than half of these patients (55.2%) died during their hospitalization, with 58% of them having AKI.

RRT was initiated in 20.4% of patients with AKI mainly for volume overload (46.6%). In one study, accumulative fluid balance was also an independent risk for developing AKI in ICU patients [36].When to initiate RRT is controversial, yet around 20% of ICU patients with AKI will need RRT within the first week of their ICU admission [41], and studies showed no benefit of early vs. late initiation of RRT on decreasing mortality or hospital stay, but studies indicate that early initiation of RRT may decrease risk for metabolic acidosis [42, 43].

Although, the average length of hospital stay between the AKI group (10.3 days) and the non-AKI group (12.3 days) was not significant ( *P* = 0.07), the cost for hospital stay was statistically significant: 7571.2 USD (SD ± 11302.2) for the AKI group vs. 4736.1 USD (SD ± 5755.9) for the non-AKI group (*P* = 0.0001).

The interesting finding of shorter hospital stay for the AKI group in our study can be explained in part by the higher in-hospital mortality among this group (55.2%) and the shorter recovery of patients who were admitted for acute cardiac illnesses.

## 5. Limitations

Although our study had a retrospective design from single center, with fewer septic patients recruited during the study period compared to other studies, to our knowledge, this is the first study from Jordan to address AKI in ICU patients with such considerably large number of patients. Our study also revealed the serious and significant impact on medical care between the AKI and non-AKI groups. Future, prospective and multicenter studies are needed to address the demographic differences at a national level and to investigate if this can affect the risk for developing AKI in the patients from our region.

## 6. Conclusion

AKI in ICU is a significant cause for prolonged ICU stay and increased morbidity and mortality. Recognition of AKI early in the course of admission and addressing the modifiable risk factors for AKI may allow early intervention to prevent and minimize adverse outcomes and complications leading to decrease morbidity and mortality caused by AKI in ICU patients.

Prospective, regional, and national studies are needed for better assessment of the incidence of AKI and better understanding and identification of risk factors, and this may be used to modify ICU policies, with the goal to improve ICU care, to lower mortality and morbidity, and minimize medical care costs.

## Figures and Tables

**Table 1:** Baseline characteristics.

Variable	% (*N*)
Age, mean (±SD)	54.3 (±SD 20.8)
*Gender*	
Male	58.0% (1,431)
Female	42.0% (1,037)
*Comorbidities*	
Diabetes mellitus	45.9% (1,133)
Hypertension	45.7% (1,127)
Ischemic heart disease	13.3% (327)
Congestive heart failure	6.7% (164)
Peripheral vascular disease	3.5% (87)
Cerebrovascular accident	6.9% (169)
Cancer	10.5% (258)
*Drugs*	
Angiotensin converting enzyme inhibitors/Angiotensin II receptor blockers	35.8% (884)
Nonsteroidal anti-inflammatory drugs	8.5% (210)
Contrast	15.7% (388)
*Severity index*	
APACHE II score, mean (±SD)	11.5 (±SD 3.95)
*Mortality*	
At the time of discharge	55.2% (482)
30 days after discharge	1.4% (32)

**Table 2:** Major causes of admission.

*Respiratory*	342 (19.2%)
Respiratory failure	4.1%
Severe pneumonia	11.6%
Pulmonary embolism	2.8%
Bronchial asthma	0.7%

*Cardiology*	194 (10.9%)
Acute coronary syndrome	6.2%
Arrhythmia	2.2%
Cardiac arrest	0.8%
Hypertensive emergency	1.7%

*Neurology*	350 (19.7%)
Hemorrhagic stroke	10.7%
Ischemic stroke	6.2%
Seizure	2.8%

*Gastrointestinal*	193 (10.9%)
Liver cirrhosis	0.9%
Upper gastrointestinal bleeding	5.8%
Abdominal pain	4.2%

*Endocrinology*	124 (7.0%)
Diabetic ketoacidosis	6.1%
Hypoglycemia	0.9%

*Sepsis*	228 (12.8%)
*Electrolyte disorders*	167 (10.6%)
*Malignancy*	180 (10.1%)

**Table 3:** Baseline characteristics by AKI.

Variable	AKI (+)	AKI (−)	*P* value
*Age, mean (± SD)*	60.3 (SD 19.1)	51.8 (SD 21.0)	**0.0001**
<60	289 (40.5%)	893 (58.7%)	
60–69	151 (21.1%)	234 (15.2%)	
>70	274 (38.4%)	401 (26.1%)	

*Sex*			
Male	408 (30.7%)	920 (69.3%)	0.29
Female	314 (32.8%)	643 (67.2%)	

*Comorbidities*			
Diabetes mellitus	344 (32.5%)	715 (67.5%)	0.15
Hypertension	366 (34.9%)	682 (65.1%)	**0.001**
Ischemic heart disease	98 (32.6%)	203 (67.4%)	0.21
Cerebrovascular accident	56 (37.6%)	93 (62.4%)	0.11
Congestive heart failure	54 (35.1%)	100 (64.9%)	0.46
Peripheral vascular disease	31 (39.7%)	47 (60.3%)	0.14
Cancer	94 (38.8%)	148 (61.2%)	**0.02**

*Drugs*			
Angiotensin converting enzyme inhibitors/Angiotensin II receptor blockers	287 (35.2%)	529 (64.8%)	**0.005**
Nonsteroidal anti-inflammatory drugs	59 (29.5%)	141 (70.5%)	0.25
Contrast	114 (31.5%)	248 (68.5%)	0.74

*Severity index*			
APACHE II	12.2 (±SD4.1)	10.7 (±SD 3.8)	**0.0001**

*Mortality*			
At the time of discharge	290 (58%)	192 (51.3%)	**0.05**
30 days after discharge	23 (1.0%)	9 (0.4%)	**0.03**

## Data Availability

The datasets used and/or analyzed during the current study are available from the corresponding author upon reasonable request.

## References

[1] Chertow G. M., Burdick E., Honour M., Bonventre J. V., Bates D. W. (2005). Acute kidney injury, mortality, length of stay, and costs in hospitalized patients. *Journal of the American Society of Nephrology*.

[2] Priyamvada P., Jayasurya R., Shankar V., Parameswaran S. (2018). Epidemiology and outcomes of acute kidney injury in critically ill: experience from a tertiary care center. *Indian Journal of Nephrology*.

[3] Santos R. P. D., Carvalho A. R. S., Peres L. A. B., Ronco C., Macedo E. (2019). An epidemiologic overview of acute kidney injury in intensive care units. *Revista da Associação Médica Brasileira*.

[4] Jiang L., Zhu Y., Zhu Y. (2019). Epidemiology of acute kidney injury in intensive care units in Beijing: the multi-center BAKIT study. *BMC Nephrology*.

[5] dos Santos R. P., Carvalho A. R. D. S., Peres L. A. B. (2019). Incidence and risk factors of acute kidney injury in critically ill patients from a single centre in Brazil: a retrospective cohort analysis. *Scientific Reports*.

[6] Forni L. G., Darmon M., Ostermann M. (2017). Renal recovery after acute kidney injury. *Intensive Care Medicine*.

[7] Rubin S., Orieux A., Clouzeau B. (2019). The incidence of chronic kidney disease three years after non-severe acute kidney injury in critically ill patients: a single-center cohort study. *Journal of Clinical Medicine*.

[8] Ülger F., Pehlivanlar Küçük M., Küçük A. O. (2018). Evaluation of acute kidney injury (AKI) with RIFLE, AKIN, CK, and KDIGO in critically ill trauma patients. *European Journal of Trauma and Emergency Surgery*.

[9] Hoste E. A., Clermont G., Kersten A. (2006). RIFLE criteria for acute kidney injury are associated with hospital mortality in critically ill patients: a cohort analysis. *Critical Care*.

[10] Mehta R. L., Kellum J. A., Shah S. V. (2007). Acute kidney injury network: report of an initiative to improve outcomes in acute kidney injury. *Critical Care*.

[11] Khwaja A. (2012). KDIGO clinical practice guidelines for acute kidney injury. *Nephron*.

[12] Waikar S. S., Bonventre J. V. (2009). Creatinine kinetics and the definition of acute kidney injury. *Journal of the American Society of Nephrology*.

[13] Hashemian S. M., Jamaati H., Farzanegan Bidgoli B. (2016). Outcome of acute kidney injury in critical care unit, based on AKI network. *Tanaffos*.

[14] Zeng X., McMahon G. M., Brunelli S. M., Bates D. W., Waikar S. S. (2014). Incidence, outcomes, and comparisons across definitions of AKI in hospitalized individuals. *Clinical Journal of the American Society of Nephrology*.

[15] Hoste E. A. J., Kellum J. A., Selby N. M. (2018). Global epidemiology and outcomes of acute kidney injury. *Nature Reviews Nephrology*.

[16] Macedo E., Garcia-Garcia G., Mehta R. L., Rocco M. V. (2019). International society of Nephrology 0 by 25 project: lessons learned. *Annals of Nutrition and Metabolism*.

[17] Singbartl K., Kellum J. A. (2012). AKI in the ICU: definition, epidemiology, risk stratification, and outcomes. *Kidney International*.

[18] Pakula A. M., Skinner R. A. (2016). Acute kidney injury in the critically ill patient. *Journal of Intensive Care Medicine*.

[19] Xiong J., Tang X., Hu Z., Nie L., Wang Y., Zhao J. (2015). The RIFLE versus AKIN classification for incidence and mortality of acute kidney injury in critical ill patients: a meta-analysis. *Scientific Reports*.

[20] Rodrigo E., Suberviola B., Albines Z. (2016). Comparación de los sistemas de clasificación del fracaso renal agudo en la sepsis. *Nefrología*.

[21] Koeze J., Keus F., Dieperink W., van der Horst I. C. C., Zijlstra J. G., van Meurs M. (2017). Incidence, timing and outcome of AKI in critically ill patients varies with the definition used and the addition of urine output criteria. *BMC Nephrology*.

[22] Luo X., Jiang L., Du B., Wen Y., Wang M., Xi X. (2014). A comparison of different diagnostic criteria of acute kidney injury in critically ill patients. *Critical Care*.

[23] Jiang F., Chen Y. H., Liang X. L. (2011). The sensitivity and accuracy of RIFLE and AKIN criteria for acute kidney injury diagnosis in intensive care unit patients. *Zhongguo Wei Zhong Bing Ji Jiu Yi Xue*.

[24] Allen J. C., Gardner D. S., Skinner H., Harvey D., Sharman A., Devonald M. A. J. (2020). Definition of hourly urine output influences reported incidence and staging of acute kidney injury. *BMC Nephrology*.

[25] Ratanarat R., Skulratanasak P., Tangkawattanakul N., Hantaweepant C. (2013). Clinical accuracy of RIFLE and acute kidney injury network (AKIN) criteria for predicting hospital mortality in critically ill patients with multi-organ dysfunction syndrome. *Journal of the Medical Association of Thailand*.

[26] Macedo E., Malhotra R., Claure-Del Granado R., Fedullo P., Mehta R. L. (2011). Defining urine output criterion for acute kidney injury in critically ill patients. *Nephrology Dialysis Transplantation*.

[27] Yue J. F., Wu D. W., Li C. (2011). Use of the AKIN criteria to assess the incidence of acute renal injury, outcome and prognostic factors of ICU mortality in critically ill patients. *Zhonghua Yi Xue Za Zhi*.

[28] Zhou J., Yang L., Zhang K., Liu Y., Fu P. (2012). Risk factors for the prognosis of acute kidney injury under the acute kidney injury network definition: a retrospective, multicenter study in critically ill patients. *Nephrology*.

[29] Zhang Q., Fei Y., Jiang L. (2019). Risk factors for mortality in intensive care unit patients with sepsis combined with acute kidney injury after continuous renal replacement therapy: secondary analysis of the data from a multicenter observational study. *Zhonghua Wei Zhong Bing Ji Jiu Yi Xue*.

[30] Pinheiro K. H. E., Azêdo F. A., Areco K. C. N., Laranja S. M. R. (2019). Risk factors and mortality in patients with sepsis, septic and non septic acute kidney injury in ICU. *Brazilian Journal of Nephrology*.

[31] Papadimitriou-Olivgeris M., Assimakopoulos S. F., Kolonitsiou F. (2019). Risk factors for acute kidney injury in critically ill patients with bacteraemia by carbapenem non-susceptible gram negative bacteria. *Le Infezioni in Medicina*.

[32] Montomoli J., Donati A., Ince C. (2019). Acute kidney injury and fluid resuscitation in septic patients: are we protecting the kidney?. *Nephron*.

[33] Ralib A., Nanyan S., Ramly N., Har L., Cheng T., Mat Nor M. (2018). Acute kidney injury in Malaysian intensive care setting: incidences, risk factors, and outcome. *Indian Journal of Critical Care Medicine*.

[34] Mohammadi Kebar S., Hosseini Nia S., Maleki N., Sharghi A., Sheshgelani A. (2018). The incidence rate, risk factors and clinical outcome of acute kidney injury in critical patients. *Iran Journal of Public Health*.

[35] Truche A. S., Ragey S. P., Souweine B. (2018). ICU survival and need of renal replacement therapy with respect to AKI duration in critically ill patients. *Annals of Intensive Care*.

[36] Zhang J., Crichton S., Dixon A., Seylanova N., Peng Z. Y., Ostermann M. (2019). Cumulative fluid accumulation is associated with the development of acute kidney injury and non-recovery of renal function: a retrospective analysis. *Critical Care*.

[37] Søvik S., Isachsen M. S., Nordhuus K. M. (2019). Acute kidney injury in trauma patients admitted to the ICU: a systematic review and meta-analysis. *Intensive Care Medicine*.

[38] Abd ElHafeez S., Tripepi G., Quinn R. (2017). Risk, predictors, and outcomes of acute kidney injury in patients admitted to intensive care units in Egypt. *Scientific Reports*.

[39] Oweis A. O., Alshelleh S. A. (2018). Incidence and outcomes of acute kidney injury in octogenarians in Jordan. *BMC Research Notes*.

[40] Alshelleh S. A., Oweis A. O., Alzoubi K. H. (2018). Acute kidney injury among nonagenarians in Jordan: a retrospective case-control study. *International Journal of Nephrology and Renovascular Disease*.

[41] Rachoin J.-S., Weisberg L. S. (2019). Renal replacement therapy in the ICU. *Critical Care Medicine*.

[42] Xiao L., Jia L., Li R., Zhang Y., Ji H., Faramand A. (2019). Early versus late initiation of renal replacement therapy for acute kidney injury in critically ill patients: a systematic review and meta-analysis. *PLoS One*.

[43] Cox K., Banerjee D. (2019). Acute renal failure in critically ill patients: current evidence-based practices. *Rhode Island Medical Journal (2013)*.

